# Autism spectrum disorder: strengthening screening for optimal outcomes

**DOI:** 10.1016/j.lansea.2026.100771

**Published:** 2026-04-16

**Authors:** The Lancet Regional Health – Southeast Asia

The Global Burden of Disease Study estimates that worldwide, 62 million people are living with autism spectrum disorder (ASD). In recent years, there has been an observed rise in measured ASD prevalence, although this could reflect increased community awareness, diagnostic capabilities, and public health response. Early diagnosis and provision of appropriate services remain key for improved outcomes among children living with ASD. For children and caregivers in low-income and middle-income countries, the path to diagnosis is often fraught with challenges; from societal barriers to system-level issues, several factors contribute to a delay in diagnosis. Despite evidence on positive outcomes from earlier institution of behavioural therapy and a recommended ideal age for screening between 18 months and 3 years, the average age of ASD diagnosis remains at 4 years. In some instances, children are not identified until they enter the formal schooling system which could impact their overall developmental trajectory. Strengthening screening is essential to improve early diagnosis.

Several challenges hinder ASD screening, such as an absence of validated locally relevant screening tools, limited numbers of trained professionals, poor clinical awareness, financial barriers, and restricted access to professional services. Systematically engaging primary care physicians in autism screening have shown promising results in high-income countries. An efficient screening strategy for low resource setting could involve strengthening engagement and awareness among paediatric and allied health-care professionals. At the community level, immunization and early childhood care programmes could be leveraged, and community health-care workers could be trained to achieve this. A toolkit developed to engage community workers in countries of Southeast Asia region has shown promising potential for improving screening.

Augmenting screening reduces delay in diagnosis, but there could also be unintended consequences of false positivity causing overclassification and parental anxiety. Given the heterogeneous manifestation of ASD and the subjective elements in evaluation, it would be prudent to consider a multilevel screening approach, in which primary screening would enrich the predictive probability of diagnosis. It is also essential to use locally developed and validated tools; although some tools have been validated in regional contexts, the linguistic diversity of the countries requires several local adaptations and validations. Screening tools also become more predictive if parents themselves express concerns and ask for evaluation. Countries could explore avenues for improving parental awareness; even knowledge on signs as simple as eye tracking might help in improving diagnosis. Given the prevailing inequities in access to even basic health services, implementing systematic ASD screening would demand considerable resources.

While several countries are still in the nascent stage of digital health technology, building low-cost, locally relevant digital ASD screening tools could be a game changer for improving access; major advantage being, reduced clinical resources and waiting time. However, development of validated, scalable digital tools remains far-fetched. At present, most tools are still under investigation; however, some have demonstrated promising results, achieving moderate accuracy through the use of video recordings, eye-tracking, and movement tracking, complemented by parent-rated activity assessments to support diagnosis. A smart phone-based tool Canva Dx is the only FDA approved application for supporting the diagnostic assessment of ASD.

The full benefits of screening are achieved only when it is integrated with appropriate diagnostic and support services. This would be contingent upon a strong referral network between pediatricians, neurologists, psychiatrists, psychologists, and other allied health professionals. Early identification not only helps parents foster acceptance but also empowers them to engage proactively in interventions, strengthening their role in supporting their child's development.

This year's World Autism Awareness Day theme of “Autism and Humanity—Every Life Has Value” embraces neurodiversity and calls for recognition of contributions by people living with autism to the community. At the same time, we are faced with the stark reality of health systems' disregard towards children with ASD and their caregivers. While early identification of ASD can transform outcomes, the most sustainable and meaningful results lie in creating families and societies in which children are free to be themselves and flourish in a loving, safe environment. At *The Lancet Regional Health – Southeast Asia* we are committed to our focus on prioritising research on neurodevelopmental disorders as highlighted in our January editorial. We welcome research focused on ASD diagnostic tools and management strategies rooted in locally relevant cultural contexts.

